# Geometric coronary constraints and anatomical feasibility of redo TAVR in Asian patients with Evolut valves: A CT-based simulation study

**DOI:** 10.1007/s12928-026-01274-2

**Published:** 2026-05-13

**Authors:** Tomoki Ochiai, Yoichi Sugiyama, Hirokazu Miyashita, Noriaki Moriyama, Koki Shishido, Futoshi Yamanaka, Yutaka Tanaka, Masato Murakami, Shigeru Saito, Kiyotaka Iwasaki

**Affiliations:** 1 Department of Cardiology, Shonan Kamakura General Hospital, Kamakura, Kanagawa Japan; 2 Cooperative Major in Advanced Biomedical Sciences, Joint Graduate School of Tokyo, Women’s Medical University and Waseda University, Waseda University, Tokyo, 162-8480 Japan; 3 Department of Integrative Bioscience and Biomedical Engineering, Graduate School of Advanced Science and Engineering, Waseda University, Tokyo, Japan; 4 Department of Modern Mechanical Engineering, School of Creative Science and Engineering, Waseda University, Tokyo, Japan; 5 Institute for Medical Regulatory Science, Waseda University, Tokyo, Japan; 6 Waseda Research Institute for Science and Engineering, Waseda University, Tokyo, Japan

**Keywords:** Aortic stenosis, Coronary obstruction, Transcatheter aortic valve replacement, Valve-in-valve

## Abstract

Redo transcatheter aortic valve replacement (TAVR) when implanting a second transcatheter valve within a tall-frame self-expanding valve can be challenging because of unfavorable coronary-related anatomy. However, computed tomography (CT)-based data in Asian patients with smaller aortic root dimensions remain limited.This study aimed to evaluate the CT-based geometric coronary constraints by simulating implantation of a balloon-expandable SAPIEN 3 (S3) within a previously implanted self-expanding Evolut valve in Asian patients. Consecutive patients who underwent TAVR using Evolut at a single center between May 2021 and May 2023 and had post-procedural CT were analyzed. Virtual S3-in-Evolut implantation was simulated at three depths (node 4: low; node 5: intermediate; node 6: high). Geometric coronary constraint was assessed based on the relationship between the neo-skirt plane and coronary ostia and the valve-to-aorta distance. Among 113 patients, the distribution of geometric coronary constraint differed by simulated S3 implantation depth. With high S3 implantation (node 6), severe, intermediate, and low constraint were observed in 63.7%, 23.9%, and 12.4% of patients, respectively; with intermediate implantation (node 5), in 31.0%, 29.2%, and 39.8%; and with low implantation (node 4), in 3.5%, 16.8%, and 79.6%. In multivariable analysis, smaller sino-tubular junction diameter was independently associated with severe geometric coronary constraint.Geometric coronary constraints that may affect coronary access after redo TAVR with S3-in-Evolut configurations depend on the implantation depth of both the index Evolut and the simulated S3. However, the hemodynamic significance and clinical impact of these geometric findings require validation through flow studies.

**Graphical Abstract:**

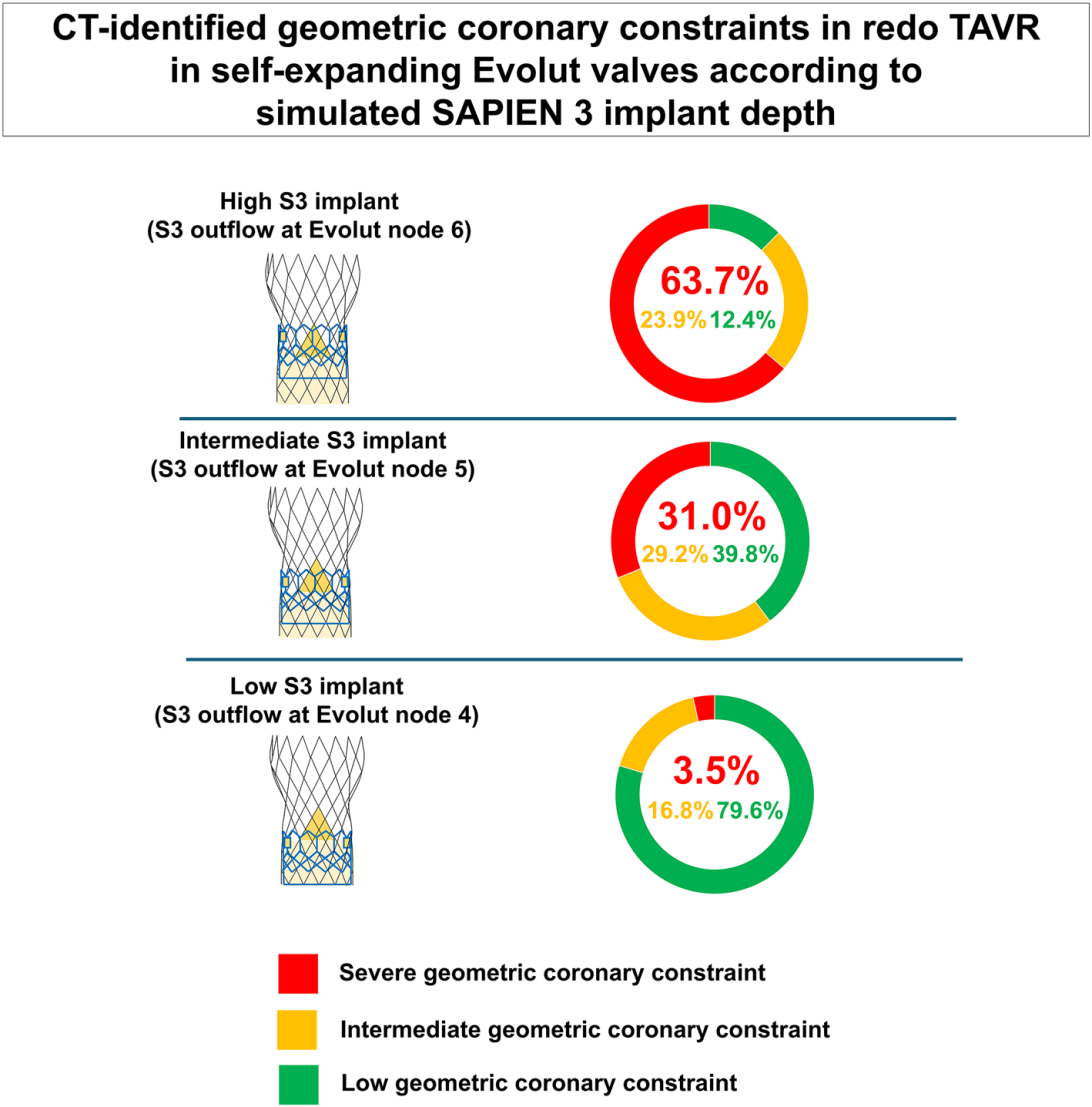

**Supplementary Information:**

The online version contains supplementary material available at 10.1007/s12928-026-01274-2.

## Introduction

Transcatheter aortic valve replacement (TAVR) is increasingly used to treat younger patients with lower surgical risk and longer life expectancy. There is a significant body of data demonstrating the durability of transcatheter aortic valves (TAVs) up to 7 years [1–3]. However, like surgical aortic bioprostheses, structural valve degeneration of TAVs can occur over time and may eventually lead to repeat intervention [4, 5]. In these cases, implantation of a second TAV inside a failed TAV (TAV-in-TAV) is a therapeutic option and the number of TAV-in-TAV procedures is expected to grow [6].

A major concern of a TAV-in-TAV procedure is that the leaflets of the first TAV would form a neo-skirt-covered stent when tilted up by a second TAV, which directly or indirectly obstructs the coronary ostium. This risk is increased with tall-frame supra-annular self-expanding valves (SEV), due to the higher leaflet position creating a taller leaflet neo-skirt [7, 8].

Previous studies conducted primarily in the United States have reported that the potential geometric coronary constraint during redo TAVR, simulated by implanting a balloon-expandable SAPIEN 3 (S3) valve (Edwards Lifesciences, Irvine, California) within an Evolut (Medtronic, Minneapolis, Minnesota) SEV, ranged from 46% to 75% in the context of high S3 implantation [7, 9]. However, comprehensive post-TAVR computed tomography (CT) assessments stratifying the severity of geometric coronary constraint by initial Evolut implantation depth and subsequent S3 implant position remain limited. Furthermore, although prior studies have shown that smaller aortic valve complexes are associated with severe geometric coronary constraint in TAV-in-TAV procedures, no data are currently available regarding this risk among Asian patients, who typically exhibit smaller aortic root anatomy [7, 9].

Therefore, we sought to utilize post-TAVR CT analysis to evaluate geometric coronary constraints and anatomical feasibility in TAV-in-TAV by simulating implantation of a S3 within Evolut SEV in various initial Evolut implant depths and subsequent S3 implant positions.

## Methods

### Study population

We screened consecutive patients with severe aortic stenosis who underwent TAVR using the Evolut TAV at Shonan Kamakura General Hospital between May 2021 and May 2023. The decision to proceed with TAVR was made with the consensus of a dedicated heart team including experienced clinical and interventional cardiologists and cardiothoracic surgeons. The selection of the type and size of TAV were at the discretion of the treating physician. For the purpose of this study, patients without electrocardiography (ECG)-gated contrast-enhanced post-TAVR CT data, those with poor CT image quality, those who underwent valve-in-valve TAVR, or those with TAV embolization were excluded from the analysis. This study was approved by the Institutional Review Board of the Tokushukai Group Ethical Committee (approval number: TGE02332-024).

## CT image analysis

### Pre-TAVR CT analysis

Details of the CT imaging acquisition protocol and processing have been previously reported [10]. For annulus, Sinus of Valsalva (SOV), sinotubular junction (STJ), and atrioventricular dimensions, analysis was performed according to the current guideline [11]. The STJ height and coronary height were measured from the annular plane to the lowest point of STJ, and inferior border of each coronary ostium in a stretched multiplanar image, respectively.

## Second TAV sizing and positioning

**Fig. 1:**
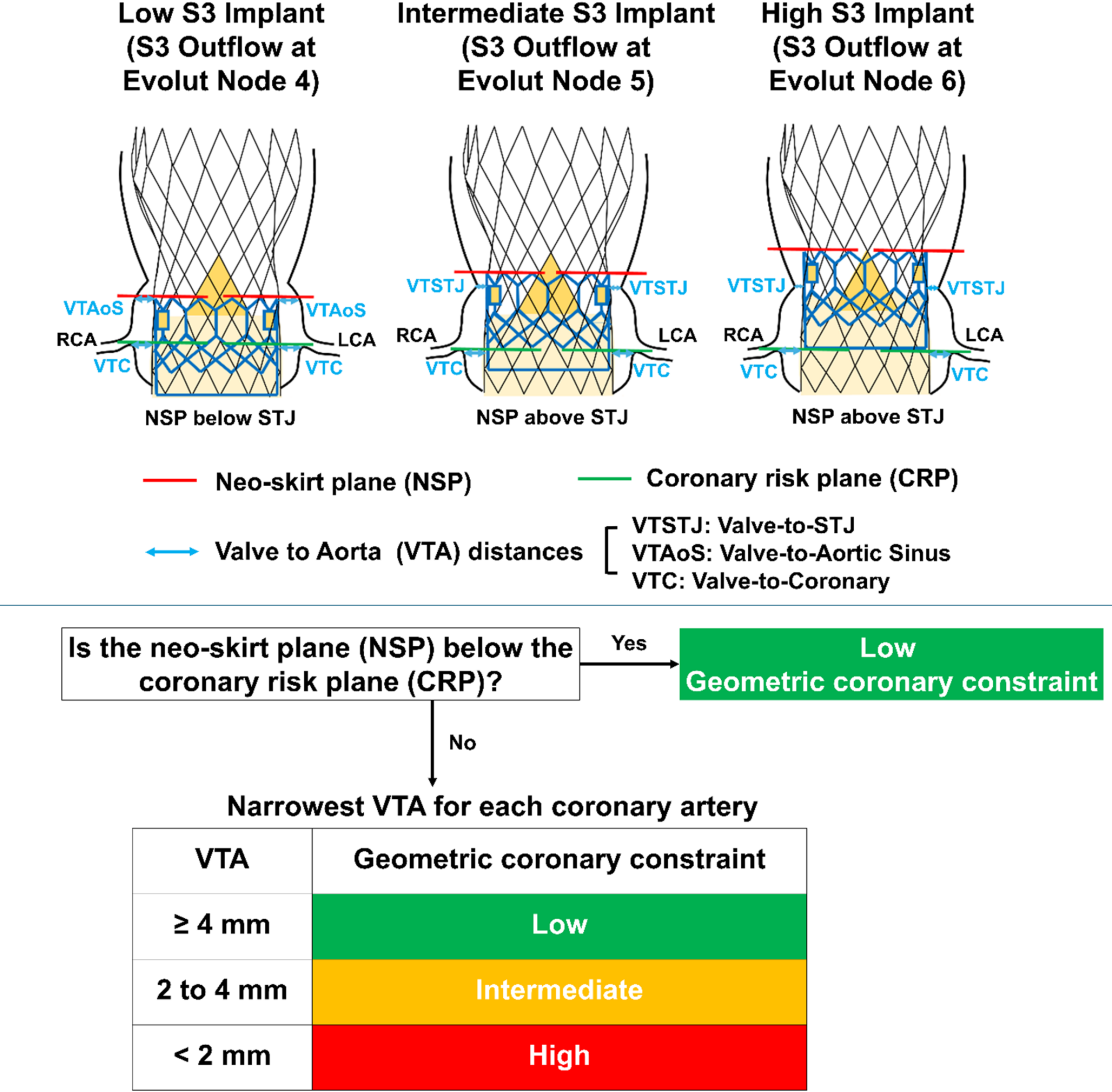
**Post-TAVR CT assessment and definition of geometric coronary constraint in redo TAVR**Measurements were determined based on the placement of virtual second S3 valve in an Evolut.CRP=coronary risk plane, CT=computed tomography, LCA=left coronary artery, NSP = neo-skirt plane, RCA=right coronary artery, S3 = SAPIEN 3, STJ=sinotubular junction, VTA=valve to aorta, VTAoS=valve to aortic sinus, VTC=valve to coronary, VTSTJ=valve to STJ, TAVR=transcatheter aortic valve replacement

The post-TAVR CT scans were used to implant virtual S3 inside the Evolut valves. For the S3-in-Evolut implantation, the S3 size was determined based on the CT measurements of the expansion of index TAV and native aortic annular dimension. Three different S3 implantation positions (low, intermediate, and high) were evaluated. In the low implantation position, the S3 outflow was aligned with Evolut node 4, in the intermediate position with node 5, and in the high implantation position with node 6 (Fig. 1). We assumed that the index TAV leaflet would be pushed completely open by the second TAV, thereby establishing a neo-skirt and sealing the stent frame circumferentially up to the S3 outflow level after TAV-in-TAV. The neo-skirt plane (NSP) is defined as the plane at the top of the neo-skirt.

## Post-TAVR CT display settings

To delineate the TAV-frame boundary and minimize blooming artifacts, measurements were performed using a standardized default display setting of WW/WL = 1,800/800 HU [12]. We confirmed the robustness of valve-to-aorta (VTA)-related measurements by repeating the analysis under two alternative window settings (WW/WL = 1600/800 and 2000/800 HU).

### Post-TAVR CT analysis and definition

To assess CT-identified geometric coronary constraints, two reference planes were identified: the NSP and the coronary risk plane (CRP). The CRP was defined as the plane parallel to the aortic annulus at the base of each coronary ostium and was established for both the left coronary artery (LCA) and the right coronary artery (RCA). In the short-axis view, the distance between the simulated redo-TAV complex and the adjacent aortic wall was measured and defined as the VTA distance. To account for stent frame thickness and blooming artifacts inherent to CT imaging, the VTA distances were measured from the midline of the stent frame. Depending on the anatomical level, the VTA distance was assessed at three locations: (i) valve-to-coronary (VTC), (ii) valve-to-aortic sinus (VTAoS), and (iii) valve-to-STJ (VTSTJ). Each measurement was obtained for both the LCA and the RCA. Geometric coronary constraint assessment was performed using the NSP, CRP, and corresponding VTA measurements for both coronary ostia, as previously described [13]. When the NSP was positioned below the CRP, the case was classified as low geometric coronary constraint. When the NSP was located above the CRP, the narrowest of the three VTA measurements was used to further stratify the geometric constraint severity. Geometric coronary constraint severity was stratified by VTA using a three-tier framework: severe (VTA < 2.0 mm), intermediate (2.0 mm ≤ VTA < 4.0 mm), and low (VTA ≥ 4.0 mm) (Fig. 1). The overall geometric coronary constraint for each patient was determined based on the higher constraint severity classification of the two coronary arteries. To assess the robustness of classification to the choice of cutoff, we performed a multi-threshold sensitivity analysis by varying the lower VTA threshold from 1.0 to 4.0 mm in 0.5-mm increments. For each threshold, we recalculated the proportion classified as severe geometric coronary constraint and plotted the results by simulated S3 outflow level.

The implantation depth of the index Evolut valve was defined as the mean distance from the inflow edge of the TAV to the floor of the sinus of Valsalva, measured at each coronary cusp.

For patients identified as having severe geometric coronary constraint, leaflet modification techniques, such as the bioprosthetic or native aortic scallop intentional laceration to prevent iatrogenic coronary artery obstruction (BASILICA) procedure may be an option to mitigate leaflet-induced coronary obstruction [14]. However, if commissural posts of the index TAV overlap with the coronary ostia, leaflet modification may be technically unfeasible or suboptimal, as the posts may remain in front of the coronary ostia even after successful leaflet laceration, thereby compromising coronary flow. To assess this, the overlap between the first TAV commissural posts and each coronary ostium was analyzed using end-diastolic phase data. Coronary overlap involving the LCA and RCA was considered severe when the neocommissure and coronary orifice were separated by an angle of 0° to 20°, as previously described [15, 16].

## Measurement reproducibility

Two observers independently remeasured left and right VTA in a randomly selected subset of 30 cases spanning the observed VTA range. Inter-observer agreement was evaluated using intraclass correlation coefficients (ICC) with a two-way random-effects model, absolute agreement, single measures, and Bland-Altman analyses reporting the mean difference and 95% limits of agreement. The primary observer repeated the measurements after an interval of at least 14 days to assess intra-observer repeatability using the same metrics. ICC values were interpreted according to established guidelines (< 0.50 = poor, 0.50–0.75 = moderate, 0.75–0.90 = good, and > 0.90 = excellent agreement) [17].

### Statistical analysis

Data are presented as mean ± standard deviation for normally distributed continuous variables or as median and interquartile range (IQR: 25–75%) for non-normally distributed continuous variables. Categorical variables are expressed as numeric values and percentages. Comparison of continuous variables was performed using the Student’s *t*-test or Mann–Whitney U test, depending on the variable distribution. The chi-square or Fisher’s exact test was used to compare categorical variables. Left and right VTA was summarized as continuous variables by simulated S3 outflow level (node 4–6) using median and interquartile range and visualized using box plots. Univariate logistic regression modelling identified initial preprocedural clinical characteristics, CT anatomical parameters, and procedural variables associated with severe coronary constraint following S3 outflow at Evolut node 6 implantations for the overall cohort. Multivariable logistic regression was performed to identify factors independently associated with severe geometric coronary constraints in case of high S3 implants. Covariates were selected based on clinical relevance and univariate associations (*p* < 0.10). The complete results of univariate logistic regression analyses for all baseline clinical characteristics, CT anatomical parameters, and procedural variables are provided in **Supplemental Table ****S1**. To avoid multicollinearity, annulus perimeter (instead of annulus area) and mean SOV diameter (instead of SOV diameters toward the left and right coronary cusps) were selected as representative CT metrics for the final multivariable model. No other relevant multicollinearity was detected. Additionally, the optimal cut-off points for the predictors for severe geometric coronary constraint were determined on a univariate level by receiver operating characteristic (ROC) curves and Youden’s J statistic. The odds ratios (OR) with associated 95% confidence intervals (CI) and Wald p-values for the continuous variables are reported. All statistical analyses were performed using SPSS version 24.0 (SPSS Inc., Chicago, IL, USA) and R software version 3.2.2 (R Foundation for Statistical Computing, Vienna, Austria). A two-sided p value < 0.05 was considered statistically significant.

## Results

For this redo TAVR CT-based analysis, we screened 142 consecutive patients for eligibility during the study period. After excluding 29 patients according to the criteria (valve-in-valve:12, no post-TAVR contrast-enhanced CT: 10, poor CT image quality: 5, TAV embolization:2), 113 patients were included in this study and post-TAVR CT scans were analyzed for implantation of a virtual second TAV in 3 different positions.

Baseline characteristics of the study population are shown in Table 1. The median age of the study cohort was 85 years, 63.7% were female, and the median Society of Thoracic Surgeon risk score was 5.3% (3.6%−7.1%). A size 23, 26, 29, 34 mm Evolut TAV was implanted in 7.1%, 48.7%, 40.7%, and 3.5% of patients, respectively. Pre- and post-dilatation was performed in 26.5% and 1.8% of TAVR procedures, respectively.

**Table 1:** Baseline characteristics

	*N* = 113
Clinical characteristics	
Age, years	85 (82–88)
Female	72 (63.7)
BSA, m^2^	1.50 (1.35–1.61)
BMI, kg/m^2^	21.8 (19.4–23.7)
Hypertension	83 (73.5)
Diabetes mellitus	35 (31.0)
Prior PCI	19 (16.8)
Prior CABG	1 (0.9)
Atrial fibrillation	25 (22.1)
Prior pacemaker	7 (6.2)
Prior stroke	9 (8.0)
eGFR < 30 ml/min/1.73 m^2^	16 (14.2)
STS score, %	5.1 (3.6–6.9)
**Pre-TAVR Echocardiography**	
LVEF, %	64.5 (57.5–69.4)
Aortic valve area, cm^2^	0.64 (0.50–0.81)
Mean gradient, mm Hg	42.9 (37.2–54.9)
**Pre-TAVR CT**	
Annular Area, mm^2^	408.0 (368.0–471.9)
Annular Perimeter, mm	72.7 (69.5–78.5)
SOV-LCC, mm	31.0 (28.6–33.3)
SOV-RCC, mm	29.5 (27.3–31.8)
Mean SOV diameter, mm	30.2 (28.5–32.9)
Mean sinotubular junction diameter, mm	26.5 (24.7–27.9)
Left sinotubular junction height, mm	18.8 (17.1–20.1)
Right sinotubular junction height, mm	19.1 (17.3–21.3)
Left coronary height, mm	13.0 (12.0–15.0)
Right coronary height, mm	14.1 (12.4–16.0)
**TAVR procedure**	
**Evolut type**	
PRO	6 (5.3)
PRO+	90 (79.6)
FX	17 (15.0)
**Evolut size**,** mm**	
23	8 (7.1)
26	55 (48.7)
29	46 (40.7)
34	4 (3.5)
Predilatation	30 (26.5)
Postdilatation	2 (1.8)

Values are expressed as total number (percentage), or median (interquartile range).BSA=body surface area; BMI=body mass index; CABG=coronary artery bypass grafting; CT=computed tomography; LCC=left coronary cusp; LVEF=left ventricular ejection fraction; PCI: percutaneous coronary intervention; RCC=right coronary cusp; SOV=Sinus of Valsalva; STS: Society of Thoracic Surgeons; TAVR=transcatheter aortic valve replacement.

### VTA measurements

VTA decreased with higher simulated S3 implantation. Median (IQR) left VTA was 4.9 (3.8–6.5) mm at node 4, 4.1 (2.6–6.3) mm at node 5, and 2.4 (0.7–3.6) mm at node 6. Median (IQR) right VTA was 5.3 (4.2–6.3) mm at node 4, 4.3 (1.6–6.0) mm at node 5, and 2.1 (0.8–4.3) mm at node 6 (**Supplemental Figure ****S1**). Inter-observer agreement was excellent for both left and right VTA (ICCs: 0.96; 95% CI, 0.92 to 0.98, and 0.98; 95% CI, 0.96 to 0.99, respectively), with small mean differences and narrow 95% limits of agreement on Bland-Altman analyses. Intra-observer repeatability was similarly high for both left and right VTA (ICCs: 0.98; 95% CI, 0.95 to 0.99, and 0.98; 95% CI, 0.95 to 0.99, respectively), with small mean differences and narrow 95% limits of agreement on Bland-Altman analyses (**Supplemental Figure S2**).

### CT-identified geometric coronary constraint based on S3 implant depth

**Fig. 2:**
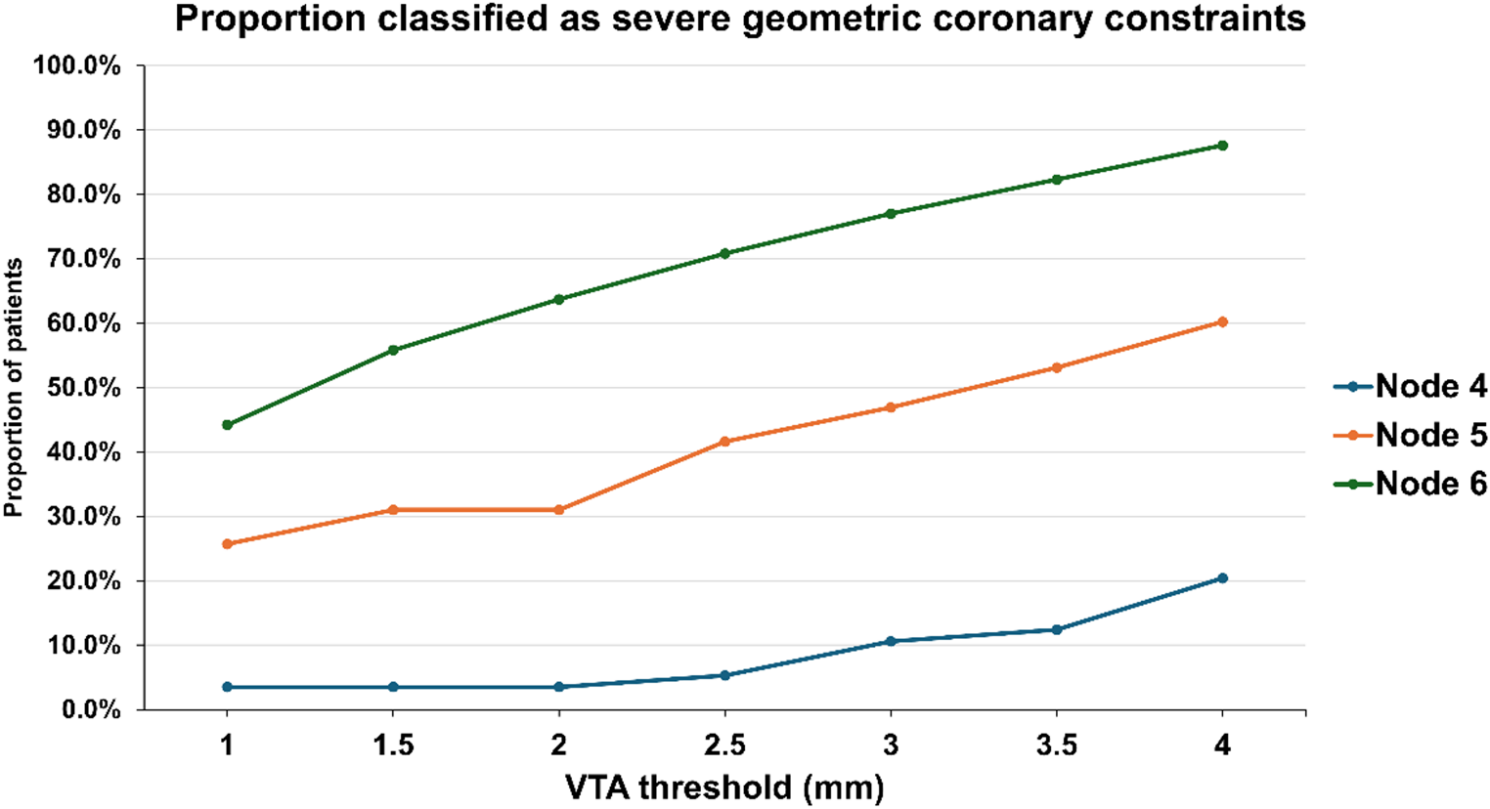
Multi-threshold sensitivity analysis for severe geometric coronary constraint according to simulated SAPIEN 3 implant depth.The x-axis shows the valve-to-aorta (VTA) threshold (1.0–4.0 mm, 0.5-mm increments) and the y-axis shows the proportion of patients classified as having severe geometric coronary constraint. Curves are stratified by simulated SAPIEN 3 (S3) implantation depth within the index Evolut valve (node 4, node 5, and node 6)

Geometric coronary constraint differed markedly by simulated S3 implantation depth. With high S3 implantation (node 6), severe, intermediate, and low constraint were observed in 63.7%, 23.9%, and 12.4% of patients, respectively. With intermediate implantation (node 5), the corresponding proportions were 31.0%, 29.2%, and 39.8%. With low implantation (node 4), severe constraint was uncommon (3.5%), whereas intermediate and low constraint accounted for 16.8% and 79.6%, respectively (**Graphical abstract**). Across VTA thresholds from 1.0 to 4.0 mm, the proportion classified as severe geometric coronary constraint increased monotonically for all simulated S3 implantation depths. Notably, the relative gradient by implantation depth remained consistent across thresholds, with node 6 showing the highest proportion of severe constraint, followed by node 5 and node 4 (Fig. 2).

#### CT-identified geometric coronary constraint based on index Evolut implant depth

**Fig. 3:**
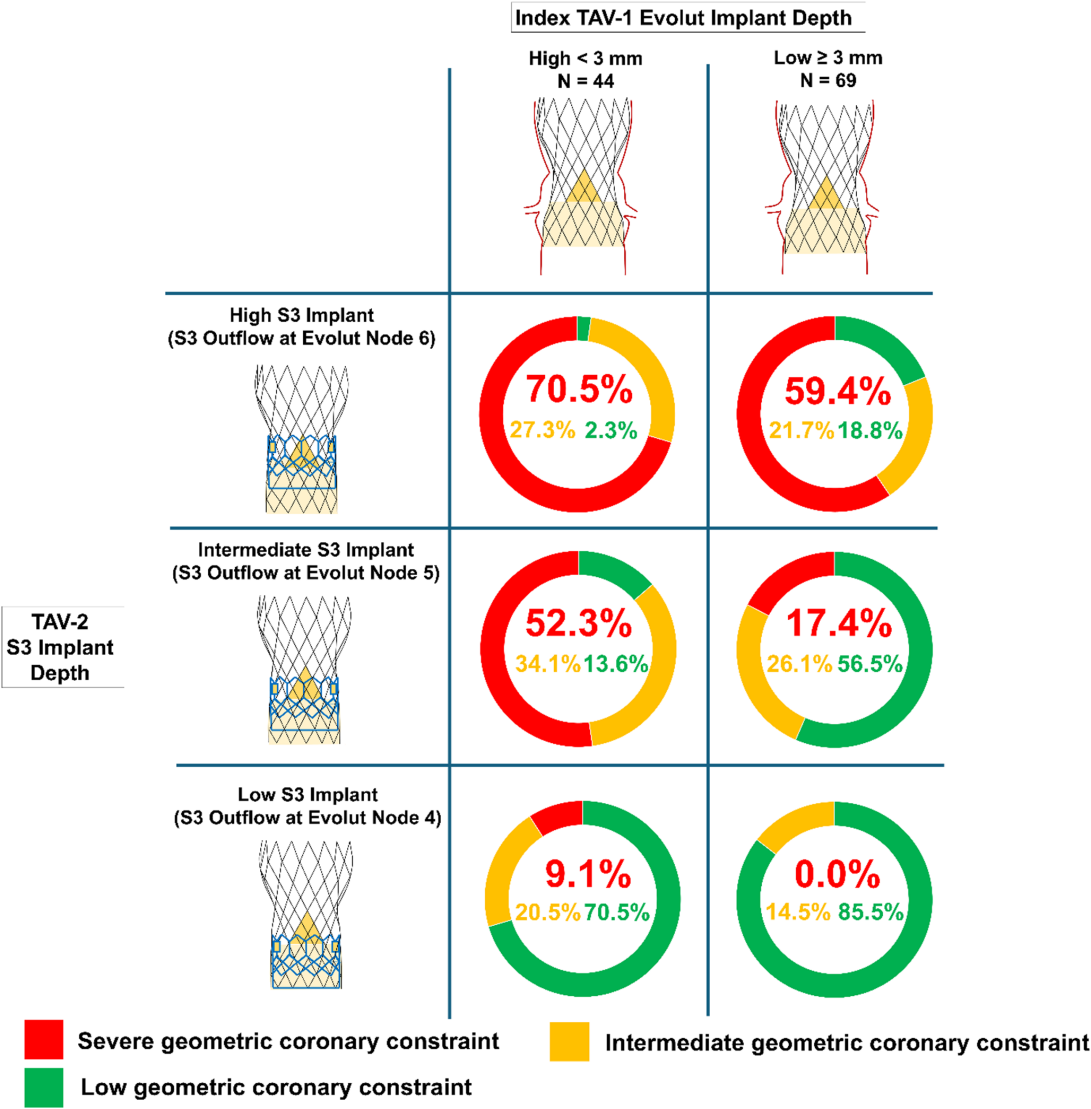
**CT-identified geometric coronary constraint according to index Evolut implantation depth and simulated SAPIEN 3 position**The likelihood of severe geometric coronary constraint following redo TAVR with S3-in-Evolut configurations was influenced by both the implantation depth of the index TAV and the simulated S3 position.S3 = SAPIEN 3, TAV=transcatheter aortic valve, TAVR= transcatheter aortic valve replacement

A high implant depth (< 3 mm) of the Evolut was observed in 38.9% of patients. Severe geometric coronary constraint became more frequent as the index Evolut was implanted more shallowly and as the simulated S3 was implanted higher. In the worst-case scenario of a shallow index Evolut implant combined with a high S3 implant, severe constraint was observed in 70.5% of patients. In the best-case scenario of a deep index Evolut implant combined with a low S3 implant, no patient met criteria for severe constraint (Fig. 3). With a low S3 implant position, severe constraint was uncommon (3.5%) irrespective of the index Evolut implant depth. The predicted constraints according to the size of the index Evolut are shown in **Supplemental Figure S3**.

### Overlap between the first TAV commissural posts and coronary ostia

In a coronary-level analysis, overlaps between the first TAV commissural posts and coronary ostium were observed in 22.0% (11 of 50) in LCA and 15.4% (8 of 52) in RCA among coronary arteries classified as having severe geometric coronary constraint with high S3 implantation (node 6) in Evolut.

### Predictors of severe geometric coronary constraint

Predictors of severe coronary constraint with a high S3 implant position (node 6) are listed in Table 2. In univariate analysis, female sex and smaller aortic root dimensions, lower STJ height, and lower coronary height were associated with a greater likelihood of severe coronary constraint. Among procedural characteristics, a shallower implantation depth of the index Evolut was associated with severe geometric coronary constraint **(**Table 2**)**. In multivariable analysis, smaller STJ diameter was independently associated with severe geometric coronary constraint (Table 3). The final multivariable logistic regression model demonstrated good discriminative performance for identifying severe geometric coronary constraint, with a C-statistic (area under the ROC curve) of 0.86. ROC analyses showed the following cut-offs for severe constraint: annulus perimeter < 72.1 mm, mean SOV diameter < 29.8 mm, mean STJ diameter < 26.7 mm, left/right STJ height < 19.1/18.6 mm, left/right coronary height < 14.9/15.1 mm, and index Evolut implantation depth < 4.0 mm.

**Table 2:** Characteristics and univariate analysis of severe geometric coronary constraint with a high SAPIEN 3 implant position (node 6) in the index Evolut

	Geometric coronary constraint		Univariate model	
	Severe (*n* = 72)	Non-severe (*n* = 41)	Odds ratio (95% CI)	*p*-value
Clinical characteristics				
Age, years	85.0 (82.0–88.0)	85.0 (82.0–89.0)	1.00 (0.93–1.08)	0.99
Male	20 (27.8)	21 (51.2)	0.37 (0.16–0.82)	0.01
BSA, m^2^	1.48 (1.35–1.59)	1.55 (1.41–1.64)	0.15 (0.02–1.49)	0.11
BMI, kg/m^2^	22.1 (19.4–25.2)	21.2 (19.3–23.4)	1.06 (0.95–1.18)	0.31
STS score, %	5.3 (3.9–6.9)	4.9 (3.5–7.1)	1.00 (0.87–1.16)	0.97
**Pre-TAVR CT**				
Annular Area, mm^2^	388.5 (347.0–445.9)	455.0 (396.7–512.5)	0.989 (0.983–0.995)	< 0.001
Annular Perimeter, mm	71.5 (67.5–75.9)	77.1 (72.5–81.6)	0.88 (0.82–0.94)	< 0.001
SOV-LCC, mm	29.7 (28.1–31.8)	33.3 (30.2–35.7)	0.74 (0.64–0.86)	< 0.001
SOV-RCC, mm	28.8 (26.7–30.4)	31.0 (29.4–33.8)	0.68 (0.57–0.81)	< 0.001
Mean SOV diameter	29.3 (27.7–31.5)	32.6 (30.2–35.5)	0.68 (0.57–0.80)	< 0.001
Mean sinotubular junction diameter, mm	25.5 (23.9–26.9)	28.2 (26.8–29.6)	0.55 (0.43–0.71)	< 0.001
Left sinotubular junction height, mm	18.1 (16.9–19.5)	19.7 (18.0–21.0)	0.77 (0.64–0.92)	0.004
Right sinotubular junction height, mm	18.3 (17.0–20.3.0.3)	20.5 (18.6–22.2)	0.73 (0.62–0.86)	< 0.001
Left coronary height, mm	13.0 (11.8–14.1)	14.0 (12.5–16.1)	0.81 (0.68–0.95)	0.01
Right coronary height, mm	13.1 (12.0–15.0)	15.9 (13.0–17.4.0.4)	0.77 (0.66–0.90)	0.001
**TAVR procedure**				
Evolut size, mm				
23	6 (8.3)	2 (4.9)	Reference	
26	42 (58.3)	13 (31.7)	1.08 (0.19–6.00)	0.93
29	24 (33.3)	22 (53.7)	0.36 (0.07–1.99)	0.24
34	0 (0.0)	4 (9.8)	NA	> 0.99
Predilatation	16 (22.2)	14 (34.1)	0.55 (0.24–1.29)	0.17
Postdilatation	2 (2.8)	0 (0.0)	NA	> 0.99
Index Evolut implantation depth*, mm	3.2 (2.7–4.0.7.0)	4.1 (2.9–5.0)	0.75 (0.57–0.98)	0.04

Values are expressed as total number (percentage), or median (interquartile range). Odds ratios and p-values were derived from univariate logistic regression. Odds ratios for continuous variables are expressed per 1-unit increase in the original scale. NA indicates not applicable. *Index Evolut implantation depth was measured in post-TAVR CT as the distance from the annular plane to the inflow edge of the Evolut frame at each coronary cusp and summarized as the mean value. Abbreviations as in Table 1.

**Table 3:** Predictors of severe geometric coronary constraint with a high SAPIEN 3 implant position (node 6) in the index Evolut

	Univariate model		Multivariable model	
	Odds ratio (95% CI)	*p*-value	Odds ratio (95% CI)	*p*-value
Clinical characteristics				
Male	0.37 (0.16–0.82)	0.01	1.53 (0.43–5.44)	0.51
**Pre-TAVR CT variables**				
Annular Perimeter, mm	0.88 (0.82–0.94)	< 0.001	0.98 (0.88–1.09)	0.65
Mean SOV diameter	0.68 (0.57–0.80)	< 0.001	0.91 (0.67–1.25)	0.57
Mean sinotubular junction diameter, mm	0.55 (0.43–0.71)	< 0.001	0.57 (0.41–0.78)	< 0.001
Left sinotubular junction height, mm	0.77 (0.64–0.92)	0.004	1.22 (0.87–1.69)	0.25
Right sinotubular junction height, mm	0.73 (0.62–0.86)	< 0.001	1.00 (0.73–1.37)	0.99
Left coronary height, mm	0.81 (0.68–0.95)	0.01	0.75 (0.57–1.00)	0.050
Right coronary height, mm	0.77 (0.66–0.90)	0.001	0.93 (0.69–1.24)	0.61
Index Evolut implantation depth*, mm	0.75 (0.57–0.98)	0.04	0.93 (0.68–1.26)	0.63

Odds ratios for continuous variables are expressed per 1-unit increase in the original scale. *Index Evolut implantation depth was measured in post-TAVR CT as the distance from the annular plane to the inflow edge of the Evolut frame at each coronary cusp and summarized as the mean value. CI=confidence interval. Other abbreviations are as in Table 1.

## Discussion

In this study, we assessed CT-identified geometric coronary constraints and anatomical feasibility for redo TAVR in Asian patients with prior self-expanding Evolut valves by simulating S3-in-Evolut configurations at different index Evolut and redo S3 implantation depths. The major findings of this study are as follows: (1) CT-identified geometric coronary constraint varied markedly by simulated S3 implantation depth within the index Evolut valve: with a high S3 position (node 6), 63.7% (*n* = 72) of patients met criteria for severe geometric coronary constraint, compared with 31.0% (*n* = 35) at the intermediate position (node 5) and 3.5% (*n* = 4) at the low position (node 4). (2) The severity of geometric coronary constraint was influenced by the implantation depths of both the index Evolut and the simulated S3. (3) Among coronary arteries classified as having severe geometric coronary constraint, coronary overlap suggesting limited anatomical feasibility for leaflet modification was observed in approximately one in five cases due to coronary–commissural misalignment. This study assessed geometric coronary constraints using static CT measurements but did not evaluate actual coronary blood flow. While narrow VTA distances may pose anatomical challenges, the hemodynamic significance remains unvalidated. Fluid dynamics analysis would be necessary to determine the relationship between VTA distance and flow velocity/pressure gradients, validate the 2 mm cutoff, and identify optimal S3 positioning. Until such validation is performed, our findings should be interpreted as hypothesis-generating data rather than definitive risk stratification.

### Optimal implantation depth of S3 in a failed Evolut TAV to preserve coronary access

Our findings demonstrated that implantation of the S3 at a lower position within the index TAV reduced the neo-skirt height, thereby lowering the likelihood of geometric coronary constraint, consistent with previous CT-based simulation studies. By using a balloon-expandable TAV inside a failed SEV, the operator can adjust the position of the S3, thereby influencing the NSP and subsequent likelihood of geometric coronary constraint. A lower implantation position, with the S3 outflow aligned at node 4, can reduce the neo-skirt height by up to 7.6 mm compared with node 6 [18]. However, a potential drawback of lower S3 implantation is the residual index TAV leaflet tissue which may overhang the S3. Although in-vitro studies of S3-in-Evolut configurations showed favorable hemodynamic outcomes across both low and high S3 positions regardless of the extent of leaflet overhung, the long-term clinical impact remain unknown [18]. This should be confirmed by further clinical studies of real TAV-in-TAV cases with longer-term follow-up. Overall, our findings highlight the importance of precise preprocedural CT assessment and individualized procedural planning, including positioning of the second TAV, to reduce the likelihood of geometric coronary constraint.

### Implantation depth of index Evolut TAV to facilitate future redo TAVR

Our study showed that deeper implantation of the index TAV was associated with a lower likelihood of geometric coronary constraint in future TAV-in-TAV. However, deeper implantation depth may increase the risk of postprocedural conduction disturbances, which are associated with higher mortality [10]. As TAVR expands to younger and lower-risk populations, determining patient-specific implantation depth becomes increasingly important to balance the competing risks of geometric coronary constraint in future TAV-in-TAV and conduction disturbances after the index procedure. This decision should be based on the anticipated need for future redo TAVR, the anatomical characteristics of the aortic valve complex, and the risk of conduction disturbances. Furthermore, anatomical characteristics and the choice or implantation depth of the index TAV may preclude future redo TAVR in some patients. The heart team should carefully determine the most appropriate initial treatment strategy (TAVR versus surgery) at the time of the first procedure. A tailored decision-making approach based on clinical and anatomical factors is essential for optimal lifetime management of patients with aortic stenosis.

### Predictors of severe geometric coronary constraint

In this study, CT-derived measurements reflecting a smaller aortic valve complex were associated with a higher likelihood of severe geometric coronary constraint in cases of high S3 implantation. Among these variables, a smaller STJ diameter emerged as the independent predictor in multivariable analysis, underscoring the critical role of the STJ in determining geometric coronary constraint after redo TAVR. It may be argued that Asian patients, who typically have smaller aortic valve complexes, are at a higher likelihood of geometric coronary constraint compared with Western populations. However, CT analysis from the Evolut low risk trial conducted primarily in the United States showed that 75% of patients were classified as severe geometric coronary constraint at a high S3 implantation position (node 6), whereas 47% and 20% were identified as severe geometric coronary constraint at intermediate (node 5) and low (node 4) implantation positions, respectively. These findings are similar to our results in Asian patients using identical methodology and a high-risk cut-off of VTA < 4 mm. Although a smaller aortic valve complex is generally associated with an increased CT-identified geometric coronary constraint in TAV-in-TAV for a given index Evolut valve size, the Evolut TAVs used in Asian patients with smaller anatomy were also correspondingly smaller than those used in prior studies involving American cohort [9]. In the majority of S3-in-Evolut configurations, a smaller Evolut TAV results in a reduced neo-skirt height, potentially mitigating the severe geometric coronary constraint [18]. Taken together, native aortic root anatomy, particularly the size of the STJ, in combination with TAV size appears to be a key determinant of geometric coronary constraint, rather than small aortic dimensions alone. Therefore, in patients with borderline annular dimensions at index TAVR, potential future coronary access difficulty and geometric coronary constraint should be carefully considered when selecting a larger TAV, particularly in the presence of a proportionally smaller STJ relative to the aortic annulus.

### Feasibility of leaflet modification procedure in TAV-in-TAV

Our analysis showed that overlap between coronary arteries and TAV neo-commissures occurred in approximately 20% of coronary arteries among classified as having severe geometric coronary constraint with high S3 implantation (node 6) in Evolut. For the Evolut TAV system, inserting the delivery system with the flush port oriented at the 3 o’clock position has been shown to enhance the success rate of achieving commissural alignment in prior studies [10, 15]. However, in this study, overlap of one or both coronary ostia with the TAV commissural posts was still observed in approximately one in five patients, even when this technique was employed. One potential explanation is that variations in aortic anatomy, including the eccentricity of coronary ostia relative to the SOV, orientation of the native aortic valve, and tortuosity of the aorta, differ substantially among patients. Future refinements in TAV and delivery system design are warranted to improve the feasibility of achieving precise commissural alignment. This finding suggests that leaflet modification strategies such as BASILICA may be technically limited in a subset of anatomies, because catheter traversal and controlled laceration may be challenging when the target leaflet region is adjacent to a neo-commissural post. In such cases, alternative management strategies should be considered, including coronary protection with a pre-positioned guidewire and standby stent, careful depth trade-offs to lower the neo-skirt relative to the coronary ostia, and heart-team discussion regarding redo-TAVR versus surgical options in anatomically unfavorable scenarios.

Given the expanding indications for TAVR in younger patients, the number of redo TAVR is expected to increase. Our findings underscore the importance of comprehensive preprocedural CT assessment to evaluate the likelihood of geometric coronary constraint by analyzing the 3-dimensional geometric interactions between the portion of the redo-TAV combination covered with the neo-skirt and aortic root. These findings suggest that even among Asian patients with smaller aortic valve complexes, careful selection of the initial Evolut implantation depth and the secondary S3 implantation position can significantly reduce severe geometric coronary constraint in TAV-in-TAV. When planning TAVR in younger low-risk patients, implantation depth and commissural alignment of the index TAV as well as the second TAV positioning during TAV-in-TAV should be carefully optimized to increase the feasibility of redo TAVR.

### Limitations

First, this study evaluated only static geometric relationships and did not assess actual coronary blood flow. The association between VTA distance and hemodynamically significant flow compromise remains unvalidated. Fluid dynamics analysis and clinical outcome studies are needed to determine whether these geometric constraints result in clinically meaningful coronary flow impairment. Second, the VTA cut-off values used for stratification, particularly the 2.0-mm threshold, are empirical and were not validated against hemodynamic assessments or clinical events. Because the relationship between VTA and functional coronary impairment is likely continuous, future studies correlating CT-based metrics with flow/hemodynamic measurements and clinical outcomes are needed to determine the optimal thresholds. Third, the neo-skirt plane definition assumes full leaflet displacement and does not incorporate patient-specific leaflet overhang or native leaflet height. Fourth, leaflet overhang or incomplete displacement could alter both hemodynamics and geometric relationship between the TAV-in-TAV complex and aortic root, particularly at lower redo TAV positions. Future work should incorporate patient-specific leaflet modeling that includes both native and index TAV leaflets and validation against flow and clinical data. Fifth, only four patients (3.5%) were classified as severe geometric coronary constraints with low S3 implantation at node 4, which limited our ability to identify predictors of geometric coronary constraints in this subgroup. Sixth, our results cannot be applied to TAV-in-TAV configurations except S3-in-Evolut. Finally, CT cannot assess the leaflets of the native aortic valve or the neointimal (pseudo endothelial) coverage of the implanted TAV device. These unresected native leaflets and neointimal tissue may persist after the index TAVR and contribute to geometric coronary constraints.

## Conclusions

In redo TAVR involving implantation of a balloon-expandable S3 valve within a prior supra-annular Evolut SEV in Asian patients, CT-identified geometric coronary constraints that may affect coronary access depend on native aortic root anatomy and the implantation depths of both the index and redo valves. Our findings suggest that preprocedural CT assessment of geometric coronary constraints may help identify patients requiring additional procedural planning for redo TAVR. However, the clinical impact of these geometric findings and optimal S3 positioning strategy require validation in actual redo-TAVR cases with clinical outcome assessment.

## Supplementary Information

Below is the link to the electronic supplementary material.

Supplementary Material 1

## Abbreviations

BMI: body mass index
BSA: body surface area
CABG: coronary artery bypass grafting
CRP: coronary risk plane
CT: computed tomography
ECG: electrocardiogram
HU: Hounsfield unit
ICC: intraclass correlation coefficient
IQR: interquartile range
LCA: left coronary artery
LCC: left coronary cusp
LVEF: left ventricular ejection fraction
NSP: neo-skirt plane
PCI: percutaneous coronary intervention
RCA: right coronary artery
RCC: right coronary cusp
S3: SAPIEN 3
SEV: self-expanding valve
SOV: sinus of Valsalva
STJ: sinotubular junction
TAV: transcatheter aortic valve
TAVR: transcatheter aortic valve replacement
