# Supplementary material for: Geometric coronary constraints and anatomical feasibility of redo TAVR in Asian patients with Evolut valves: A CT-based simulation study
Source: Cardiovasc Interv Ther. 2026 May 13;41(3):749–60. doi: 10.1007/s12928-026-01274-2 (PMC13279595; doi:10.1007/s12928-026-01274-2)

**Supplementary Materials**

**Geometric coronary constraints and anatomical feasibility of redo TAVR in Asian patients with Evolut valves: A CT-based simulation study**

Tomoki Ochiai^1,2^, Yoichi Sugiyama^1^, Hirokazu Miyashita^1^, Noriaki Moriyama^1^, Koki Shishido^1^, Futoshi Yamanaka^1^, Yutaka Tanaka^1^, Masato Murakami^1^, Shigeru Saito^1^ and Kiyotaka Iwasaki^2,3,4,5,6^

^1^Department of Cardiology, Shonan Kamakura General Hospital, Kamakura, Kanagawa, Japan

^2^Cooperative Major in Advanced Biomedical Sciences, Joint Graduate School of Tokyo Women’s Medical University and Waseda University, Waseda University, Tokyo, Japan

^3^Department of Integrative Bioscience and Biomedical Engineering, Graduate School of Advanced Science and Engineering, Waseda University, Tokyo, Japan

^4^Department of Modern Mechanical Engineering, School of Creative Science and Engineering, Waseda University, Tokyo, Japan

^5^Institute for Medical Regulatory Science, Waseda University, Tokyo, Japan

^6^Waseda Research Institute for Science and Engineering, Waseda University, Tokyo, Japan

**Table S1. Complete univariate logistic regression analysis for severe geometric coronary constraint with a high SAPIEN 3 implant position (node 6) in the index Evolut**

|  | **Univariate model** | |
| --- | --- | --- |
|  | **Odds ratio (95% CI)** | **p-value** |
| **Clinical characteristics** | | |
| Age | 1.00 (0.93-1.08) | 0.99 |
| Male | 0.37 (0.16-0.82) | 0.01 |
| BSA, m^2^ | 0.15 (0.02-1.49) | 0.11 |
| BMI, kg/m^2^ | 1.06 (0.95-1.18) | 0.31 |
| **Pre-TAVR CT** | | |
| Annular Perimeter, mm | 0.88 (0.82-0.94) | <0.001 |
| Mean SOV diameter | 0.68 (0.57-0.80) | <0.001 |
| Mean sinotubular junction diameter, mm | 0.55 (0.43-0.71) | <0.001 |
| Left sinotubular junction height, mm | 0.77 (0.64-0.92) | 0.004 |
| Right sinotubular junction height, mm | 0.73 (0.62-0.86) | <0.001 |
| Left coronary height, mm | 0.81 (0.68-0.95) | 0.01 |
| Right coronary height, mm | 0.77 (0.66-0.90) | 0.001 |
| **TAVR procedure** |  |  |
| Index Evolut implantation depth*, mm | 0.75 (0.57-0.98) | 0.04 |

Odds ratios for continuous variables are expressed per 1-unit increase in the original scale. *Index Evolut implantation depth was measured in post-TAVR CT as the distance from the annular plane to the inflow edge of the Evolut frame at each coronary cusp and summarized as the mean value. BSA=body surface area; BMI=body mass index; CI=confidence interval; CT=computed tomography; SOV=Sinus of Valsalva; TAVR=transcatheter aortic valve replacement.

**Figure S1. VTA distance by simulated SAPIEN 3 implantation depth within the index Evolut valve**

**
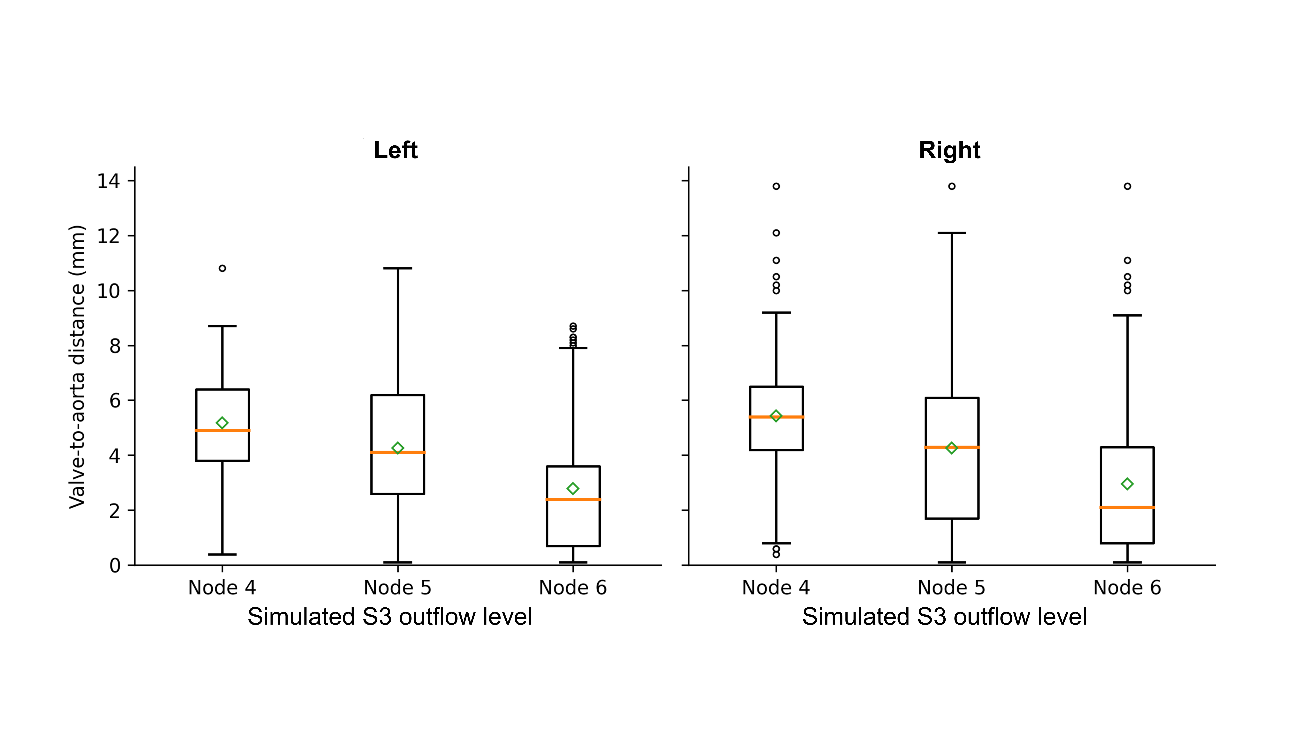
** Box plots show left and right VTA distances (mm) following virtual S3-in-Evolut implantation at three outflow positions (node 4, low; node 5, intermediate; node 6, high). SAPIEN 3=S3; VTA=valve to aorta

**Figure S2. Reproducibility of VTA measurements (Bland–Altman analysis)**

1. **Intra-observer: left VTA**

**
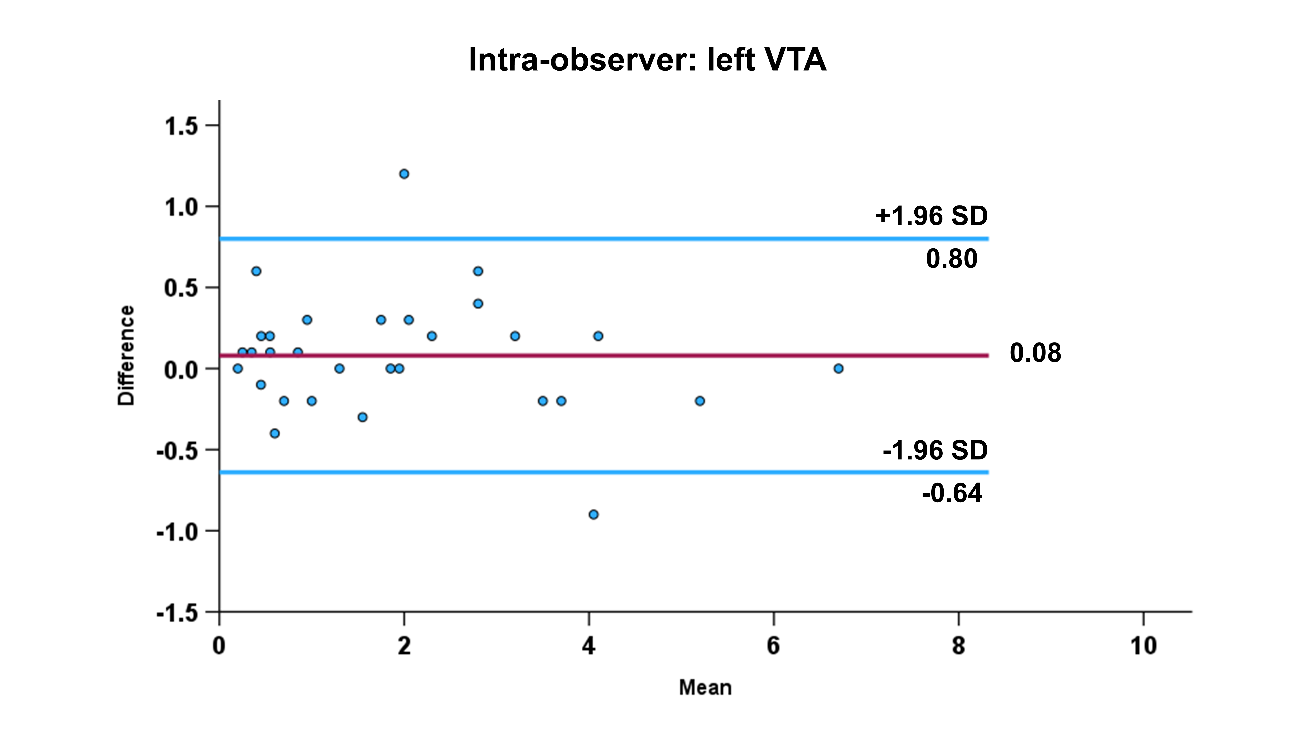
**

1. **Inter-observer: left VTA**

**
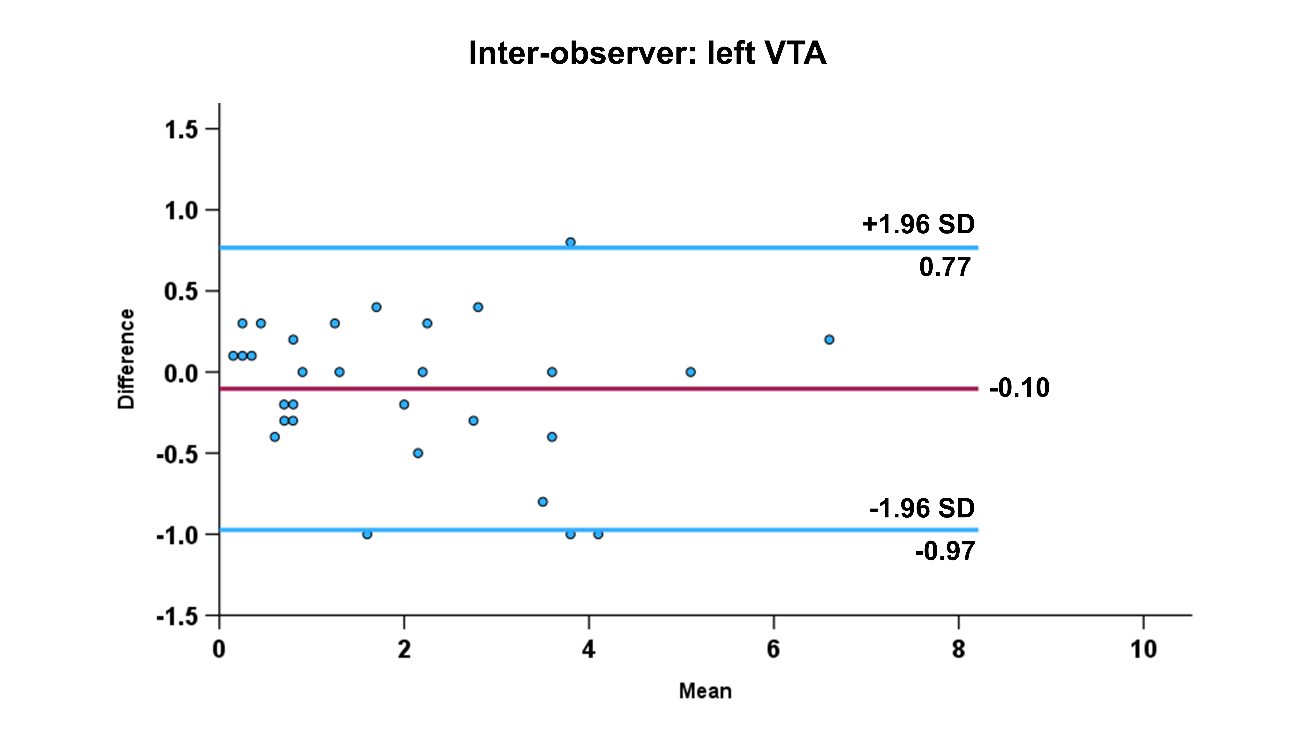
**

1. **Intra-observer: right VTA**

**
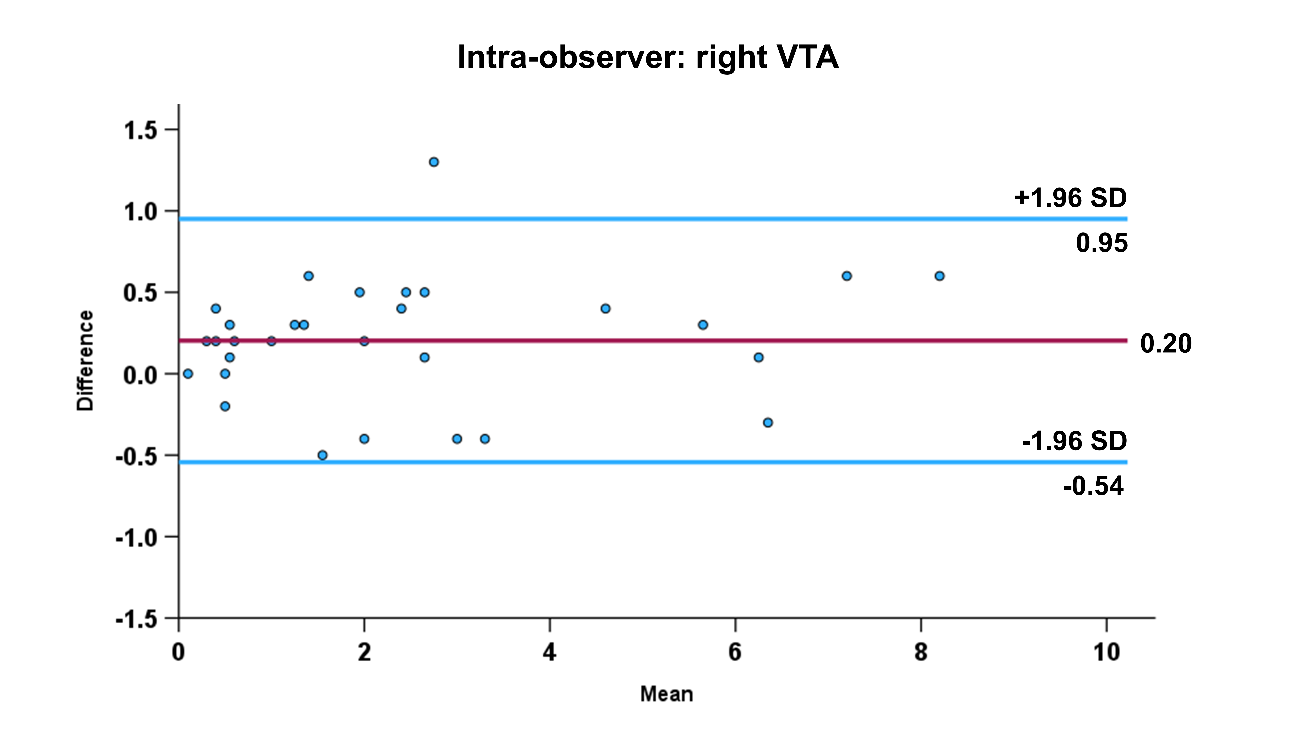
**

1. **Inter-observer: right VTA**

**
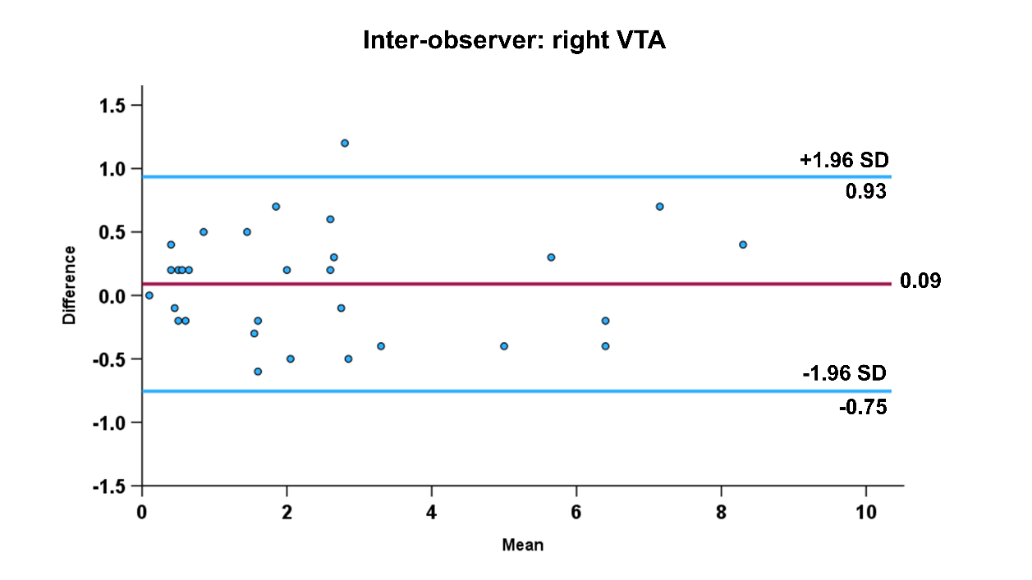
**

**Figure S3. Distribution of CT-identified geometric coronary constraint stratified by index Evolut valve size**

1. **Evolut 23 mm**


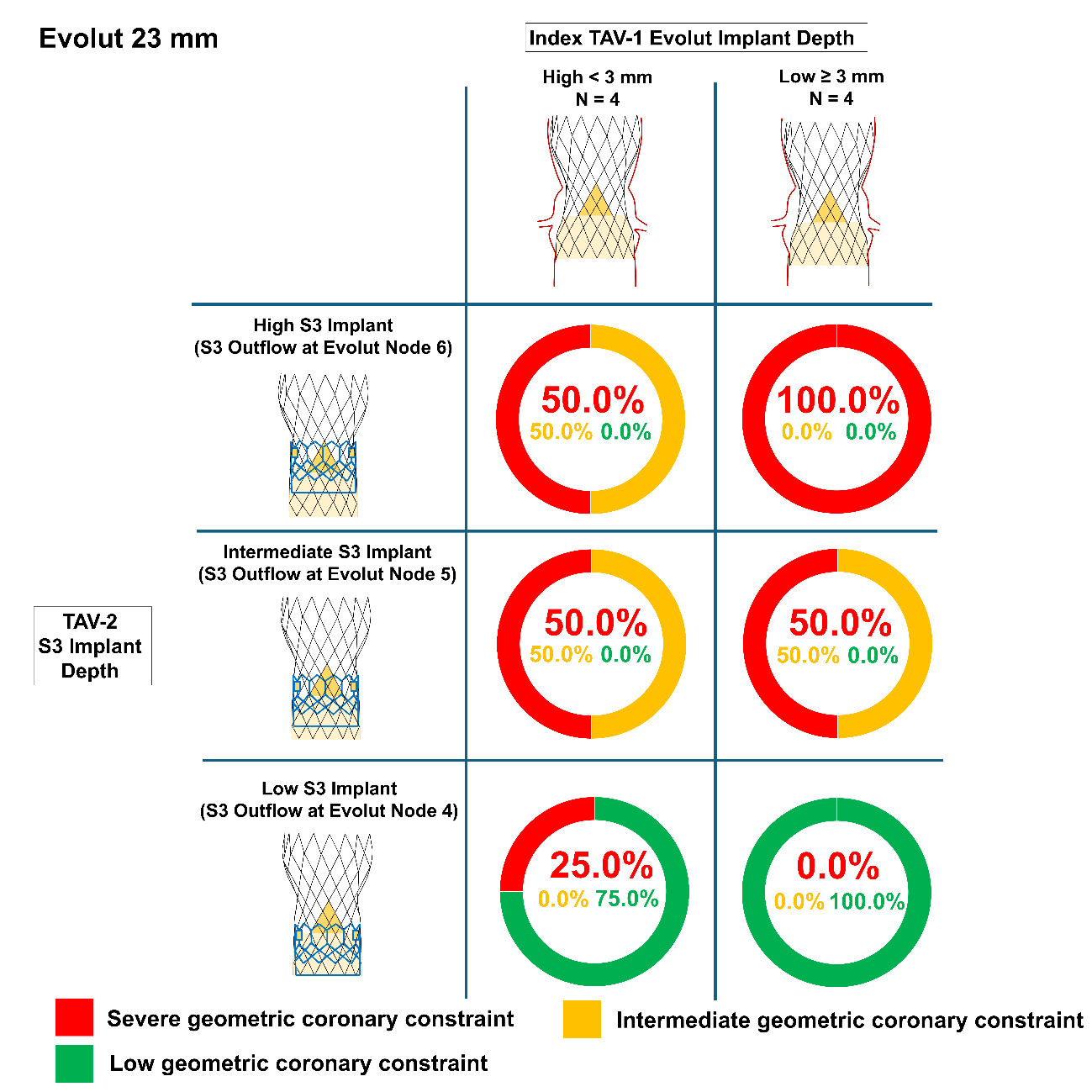


1. **Evolut 26 mm**


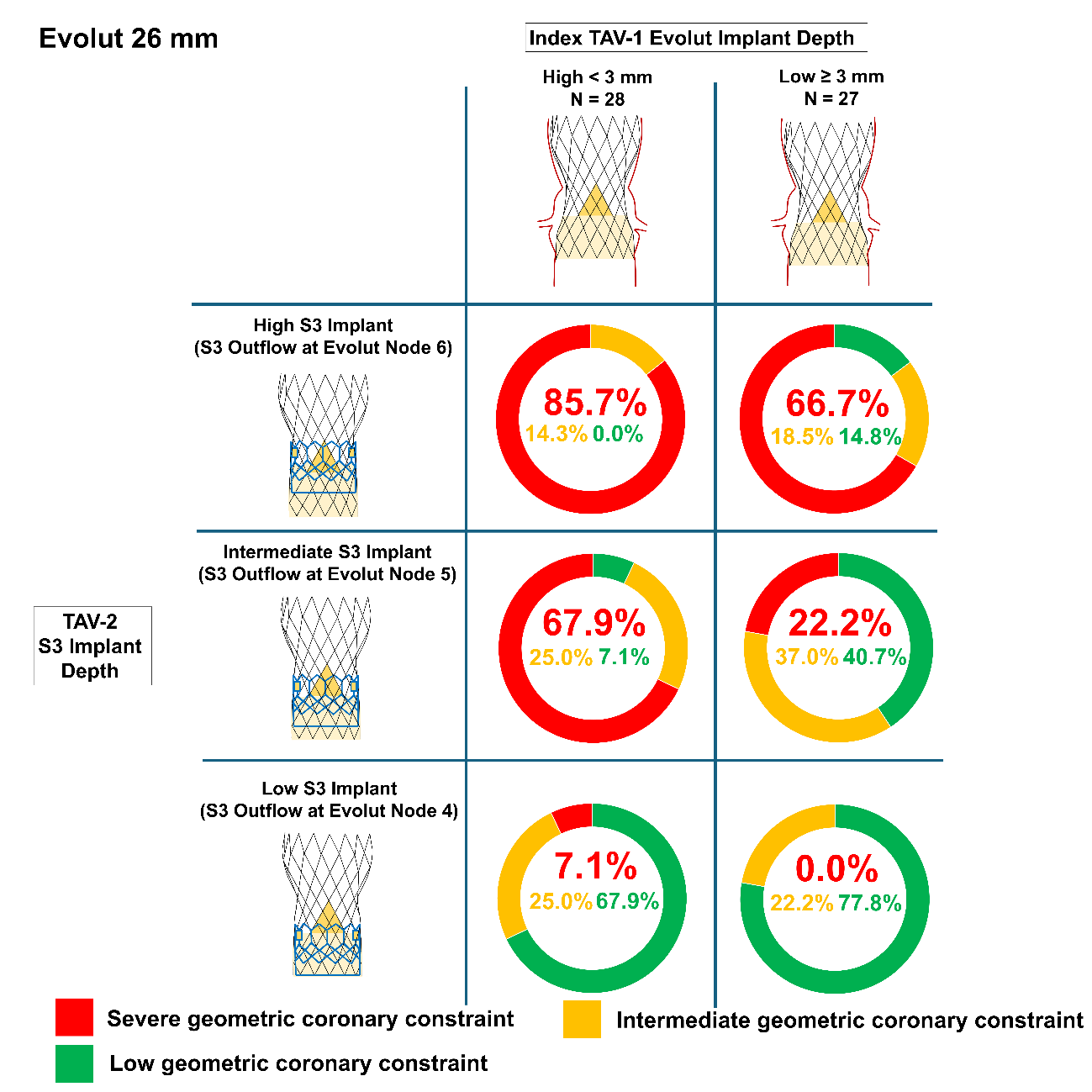


1. **Evolut 29 mm**


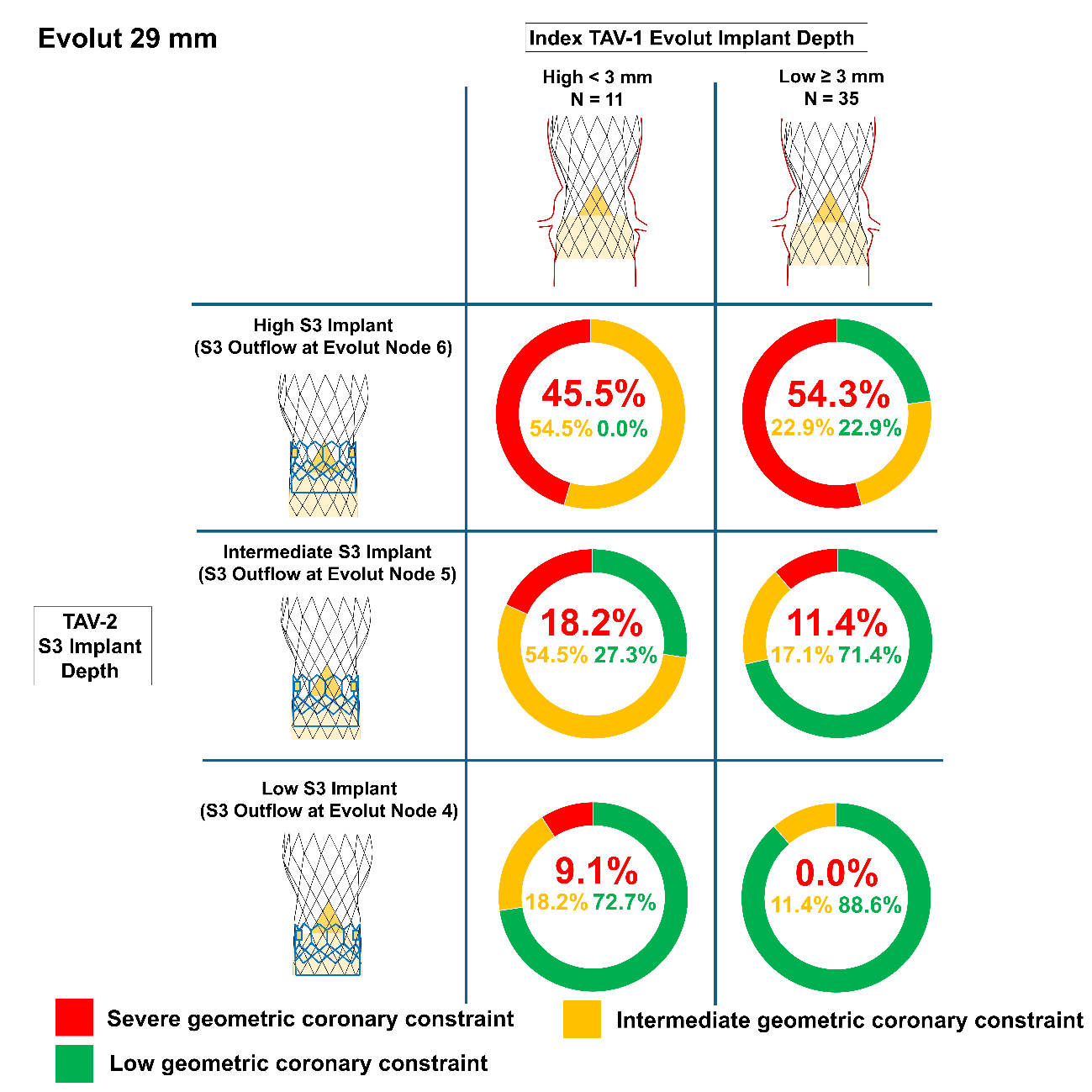


1. **Evolut 34 mm**


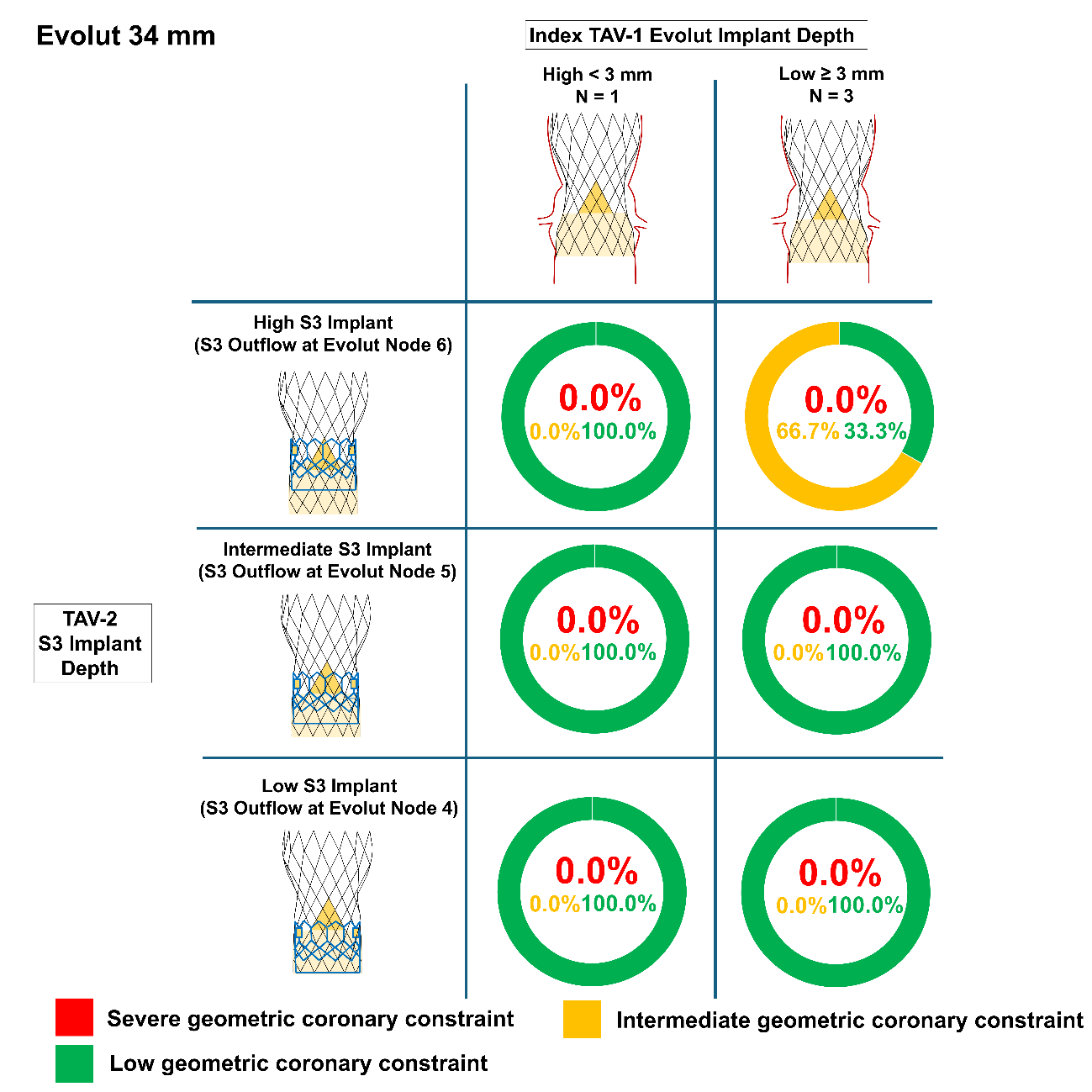

Supplement: Supplementary file 1 — Supplementary Material 1 [file 12928_2026_1274_MOESM1_ESM.docx]
